# Novel Mouse Model of Recurrent Sublethal Herpes Simplex Virus Infection Recapitulates Human Antibody Responses to Primary and Chronic Infection

**DOI:** 10.3390/vaccines12101127

**Published:** 2024-10-01

**Authors:** Aakash Mahant Mahant, Tania Jaimes Gualdron, Betsy C. Herold

**Affiliations:** 1 Departments of Microbiology-Immunology, Albert Einstein College of Medicine, Bronx, NY 10461, USA; aakash.mahant@einsteinmed.edu (A.M.M.); tania.jaimesgualdron@einsteinmed.edu (T.J.G.); 2 Departments of Pediatrics, Albert Einstein College of Medicine, Bronx, NY 10461, USA

**Keywords:** herpes simplex virus, vaccines, antibody-dependent cellular cytotoxicity, recurrent infections, mouse model

## Abstract

**Background:** Herpes simplex virus (HSV) vaccine development has been impeded by the absence of predictive preclinical models and defined correlates of immune protection. Prior candidates elicited neutralizing responses greater than natural infection but no antibody-dependent cellular cytotoxicity (ADCC) and failed to protect in clinical trials. Primary HSV infection also elicits only neutralizing responses, but ADCC and an expanded antigenic repertoire emerge over time. This evolution may contribute to the decreased frequency and severity of recurrences. To test this notion, we developed a recurrent HSV infection mouse model and evaluated changes in humoral immunity with repeated challenges. **Methods:** Mice were repeatedly infected intranasally with clinical isolates of HSV-1 or HSV-2 for four months. HSV binding IgG, neutralizing (with or without complement) and ADCC-mediating antibodies were quantified prior to each round of infection. Viral targets were assessed by western blotting. Pooled immune serum (750 μg IgG per mouse) was passively transferred into naïve wild-type or *Hvem* knockout mice 24 h prior to lethal skin challenge. **Results:** Repeated exposure to HSV-1 or HSV-2 induced an increase in total HSV-binding IgG but did not boost neutralizing titers. In contrast, ADCC-mediating responses increased significantly from the first to the fourth viral exposure (*p* < 0.01). The increase was associated with an expanded antigenic repertoire. Passive transfer of fourth round immune serum provided significant protection whereas first round serum failed to protect (*p* < 0.01). However, protection was lost when serum was transferred into *Hvem* knockout mice, which are impaired in mediating ADCC killing. **Conclusion:** This novel model recapitulates clinical responses, highlights the importance of ADCC in protecting against recurrent infection, and provides a strategy for evaluating therapeutic vaccines.

## 1. Introduction

Despite decades of research, efforts to develop an effective prophylactic or therapeutic vaccine against herpes simplex virus type 1 and/or type 2 (HSV-1 and/or HSV-2) have met with little success [1]. This reflects, in part, the size and complexity of the viruses, multiple immune evasion strategies, absence of defined correlates of immune protection and lack of highly reproducible and predictive models of primary or recurrent disease. Natural immunity provides incomplete protection against recurrences, reinfection, or heterologous infection (e.g., HSV-2 infection in an HSV-1 seropositive person or vice versa). Thus, effective vaccines must elicit a stronger and/or different immune response than natural infection.

Prior vaccine candidates were primarily designed to elicit high titer neutralizing antibody (nAb) responses. However, while clinical trial results showed that neutralizing titers that exceeded the response to natural infection were elicited by the two adjuvanted subunit protein candidates that were advanced to efficacy trials (one comprised of HSV-2 glycoprotein D (gD-2) and glycoprotein B (gB-2) with the adjuvant MF59 and the other, gD-2 with the adjuvant AS04), there was no significant protection against infection or disease [2,3]. In subsequent studies, it was noted that neither of these vaccines elicited antibodies that mediated antibody-dependent cellular cytotoxicity (ADCC) [4,5]. More recently, a replication defective viral vaccine candidate, HSV-2 dl5-29, which is deleted in *U_L_5* (helicase-primase) and *U_L_29* (DNA binding protein), was evaluated in a Phase 1 safety and immunogenicity study. Preclinical studies showed that the vaccine generated high titer nAbs as well as CD4 and CD8 T cell responses and protected against acute or recurrent genital disease when mice or guinea pigs were infected vaginally with laboratory adapted viral strains [6]. However, in the Phase 1 clinical trial, dl5-29 induced only modest CD4+ T cell response and little or no CD8+ T cell responses in seronegative individuals and did not boost nAb titers in HSV-1 or HSV-2 seropositive participants [7]. Subsequent studies showed that the vaccine elicited antibody responses to fewer epitopes in seronegative participants compared to natural infection [8].

The failure of the traditional vaginal mouse model to predict clinical trial outcomes highlights the need for more predictive preclinical models of both primary and recurrent infections. We previously introduced several changes to models of primary infection by challenging mice with lethal doses of clinical isolates of HSV-1 or HSV-2 either vaginally (female) or on the skin (female and male) [1,9,10,11]. Results recapitulated the outcome of the gD-2AS04 clinical trials. The gD-2AS04 vaccine elicited neutralizing responses in mice comparable to those elicited in study participants but little or no ADCC response [11,12]. Moreover, neither active nor passive immunization protected mice from lethal skin or vaginal challenges with clinical viral isolates. In contrast, vaccination of male or female mice with ΔgD-2, an engineered HSV-2 virus deleted in gD and thus restricted to a single cycle, was fully protective against multiple clinical viral isolates following either active or passive immunization in these models. This candidate vaccine, which is in preclinical development, elicits a predominant ADCC response, and the importance of these ADCC mediating antibodies was confirmed by the loss of active or passive protection in Fc gamma receptor IV (FcγRIV) or herpes virus entry mediator (*Hvem*) knockout mice [12]. FcγRIV is the primary mediator of ADCC in mice and HVEM, a member of the tumor necrosis factor receptor family, is involved in generating ADCC-mediating antibodies but also plays a key role in effector cell killing [12]. These results challenge the reliance on nAbs for primary protection, highlight the importance of ADCC, and suggest that more stringent mouse models using clinical isolates may prove more predictive.

While primary infection and the subunit protein vaccines elicit only nAbs, ADCC-mediating antibodies are detected in patients who have been HSV-2 seropositive for at least 5 years [5]. These ADCC-mediating Abs target several different viral proteins but not gD [5]. Similar to the clinical observations, we previously demonstrated that sublethal intranasal infection of mice also elicited a neutralizing but little or no ADCC response [13]. We hypothesized that repeated sublethal intranasal exposures might provide a model of HSV recurrent disease and recapitulate the evolution of humoral immune responses observed clinically. To test this notion, we exposed mice monthly to clinical isolates of HSV-1 or HSV-2, quantified and characterized the antibody response and tested the immune serum for ability to passively protect naïve wild-type and *Hvem* knockout mice.

## 2. Materials and Methods

### 2.1. Cells and Virus

Vero (African green monkey kidney cells, CCL-81, American Type Culture Collection) were grown in Dulbecco’s Modified Eagle Medium (DMEM) (Thermo-Fisher Scientific, Waltham, MA, USA) supplemented with 10% fetal bovine serum (HyClone, Logan, UT, USA) and 1% penicillin-streptomycin (Thermo-Fisher Scientific, Waltham, MA, USA). HSV-1 (B^3^×1.1) and HSV-2 (4674), low passage clinical isolates, were propagated on Vero cells and the viral titers were determined by plaque assay [9].

### 2.2. Sublethal Infections and Passive Transfer Studies in Mice

The study was approved by the Institutional Animal Care and Use Committee at the Albert Einstein College of Medicine, protocol 00001161. C57BL/6J mice (female or male), purchased from Jackson Laboratory (JAX, Bar Harbor, ME, USA), were infected monthly for 4 months by intranasal instillation of 10^4^ pfu of HSV-1 (B^3^×1.1) or HSV-2 (4674). Blood was collected by retro-orbital bleeding one-week prior to each round of infection. For passive transfer studies, pooled immune serum containing 750 µg total IgG harvested from mice prior to any infection (R0) or after the first (R1) or fourth (R4) round of infection was inoculated intraperitoneally into naïve BL/6 or *Hvem* knockout mice [14]. (gift from R. Longnecker, Northwestern University) 24 h prior to skin challenge with an LD90 of HSV-1 (B^3^×1.1) or HSV-2 (4674). Mice were monitored for 14 days following challenge and scored for signs of disease as previously described [9].

### 2.3. Antibody Assays

Serum was separated, divided into aliquots and stored at −80 °C for immunological assays.

Total HSV-infected cell lysate binding, gD or gB specific IgG were measured in serum by enzyme-linked immunosorbent assays (ELISA) as previously described [5,12,15]. Briefly, plates were coated with lysates of Vero cells that had been infected for 24 h with HSV-1 or HSV-2 at a multiplicity of infection (MOI) of 0.1 pfu/cell, uninfected Vero cells as background or recombinant proteins (10 ng/well). Dilutions of mouse serum were then added to duplicate wells and incubated overnight at 4 °C and bound IgG quantified using horse radish peroxidase-conjugated secondary antibodies (Thermo Scientific, Waltham, MA, USA).

Neutralization titers were calculated by plaque-reduction assay with serum that was heat inactivated at 56 °C for 30 min without (complement-independent) or with the addition of 10% rabbit complement (complement-dependent) [16]. Plaques were counted after 48 h and the neutralization titer defined as the highest dilution resulting in a 50% reduction in plaques compared to control wells (virus incubated with media only). FcγRIV activation, a biomarker of ADCC, was measured using the mFcγRIV ADCC Reporter Bioassay (Promega, Madison, WI, USA) [9]. Fold-induction was calculated relative to luciferase activity in the absence of serum.

Western blots were performed with HSV-1 or HSV-2 infected or uninfected Vero cell lysates (10 µg per lane) as the immunogen [5]. Blots were blocked for 2 h with 5% milk in phosphate buffered saline containing 0.5% Tween-20, cut into strips and each strip incubated with 10 µL of immune serum from individual mice in blocking buffer overnight, and then with HRP-labeled anti-mouse IgG (1:500, BioRad 1706516). Blots were scanned using a ChemiDoc imaging system equipped with GelDOC200 software Image Lab 6.0.

### 2.4. Statistical Analysis

Analyses were performed using GraphPad PRISM version 10.3.1 (GraphPad Software, San Diego, CA, USA). A *p*-value of 0.05 was considered statistically significant. Results were compared by ANOVA with multiple comparison tests paired or paired two-tailed *t* tests as indicated. Survival curves were compared using the Gehan-Breslow-Wilcoxon test.

## 3. Results

**Figure 1:**
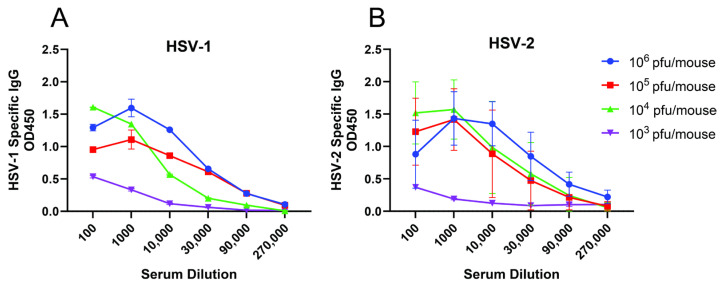
***Sublethal intranasal infections with 10^4^ pfu/mouse consistently elicits an HSV-specific antibody response***. Mice were infected intranasally with (**A**) HSV-1 (B^3^×1.1) or (**B**) HSV-2 (4674) at the indicated doses (n = 5 per group). Blood was collected one-week post-infection and serial dilutions of pooled serum from each group were assayed for HSV-specific IgG by ELISA. Results are shown as mean ± SD from duplicates as optical density at 450 nm (OD450).

***Establishment of repeated sublethal intranasal infection model*:** In prior published studies, we found that intranasal infection of mice with ~10^4^ pfu of the clinical isolates HSV-1 (B^3^×1.1) or HSV-2 (4674) consistently resulted in survival of 60–80% of mice [13]. To determine if this sublethal dose also elicited an HSV-specific antibody response, blood was collected one week post infection in mice intranasally infected with escalating doses of virus. Little or no HSV-specific IgG was detected in mice infected with 10^3^ pfu of virus, which resulted in 100% survival but the IgG response in mice infected with 10^4^ pfu (or greater) elicited a consistent IgG response (Figure 1). In contrast, low dose skin or vaginal infections yielded inconsistent survival and antibody responses.

**Figure 2:**
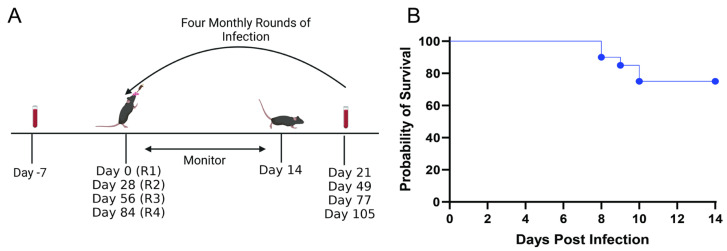
***Model of recurrent HSV infection***. (**A**) Mice were intranasally infected with 10^4^ pfu of HSV-1 (B^3^×1.1) at Day 0 and monitored for disease. Surviving mice were then reinfected monthly for a total of four rounds of infection. Blood was obtained one week prior to the first infection (Day-7, R0) and 3 weeks after each round of infection (Days 21, 49, 77, and 105) (R1–R4, respectively). (**B**) Survival of mice after the first round of infection; n = 20 (10 each in two independent experiments).

Thus, based on these findings, we selected the intranasal route of infection with 10^4^ pfu of virus to develop and characterize the humoral immune response to repeated infections. We focused first on HSV-1, as intranasal inoculation may more closely reflect the route of clinical HSV-1 exposures. Mice (6–8 weeks of age, 15 females and 5 males) were infected with 10^4^ pfu of HSV-1 intranasally and monitored for signs of disease for 14 days and then reinfected four times at monthly intervals. Blood was collected one week prior to each round of infection (Figure 2A)**.** Seventy-five percent (15/20) mice survived the first round of infection with HSV-1 (B^3^×1.1) (Figure 2B) and none succumbed to subsequent exposures. There was no difference in survival comparing males and females (11/15 female and 4/5 males).

**Figure 3:**
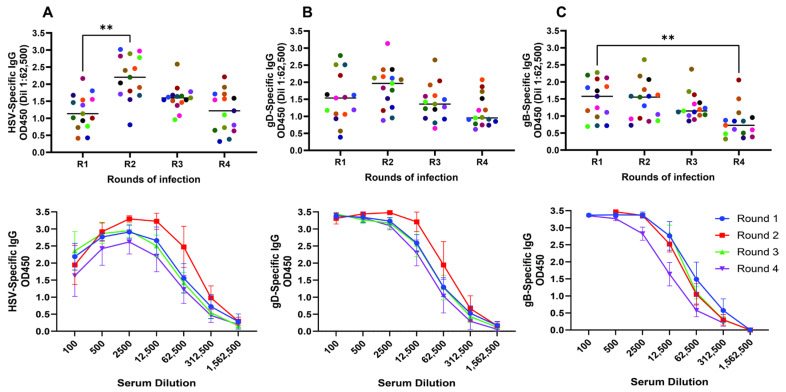
***Repeated infections transiently boost HSV-specific antibody responses***. Serum obtained 21 days after each round of infection was assayed for HSV-infected cell lysate (**A**), recombinant gD (**B**) or recombinant gB (**C**) binding by ELISA. The upper panel shows results for each individual color-coded mouse at a serum dilution of 1:62,500 (corresponding to the dilution associated with a 50% reduction in the OD450) (n = 15) and the lower panels show the full dilution curves (mean ± sd, n = 7 mice). The asterisks indicate significance relative to R1 serum (ANOVA with Freidman test for multiple comparisons) (** *p* < 0.01).

***Repeated infection boosts ADCC but not binding or neutralizing antibody responses***: Antibodies recognizing HSV-infected cell lysates, recombinant gD and gB proteins were elicited in response to primary viral infection. No HSV-specific antibodies were detected in preinfection serum (R0). The HSV-1 lysate binding IgG was transiently and significantly boosted after the second viral exposure (R2) but not subsequent exposures (R3 and R4) (Figure 3A). There was no significant increase in gD (Figure 3B) or gB (Figure 3C) binding antibodies, which decreased over time and was statistically significant for the gB-specific IgG comparing R4 and R1 (*p* < 0.01).

**Figure 4:**
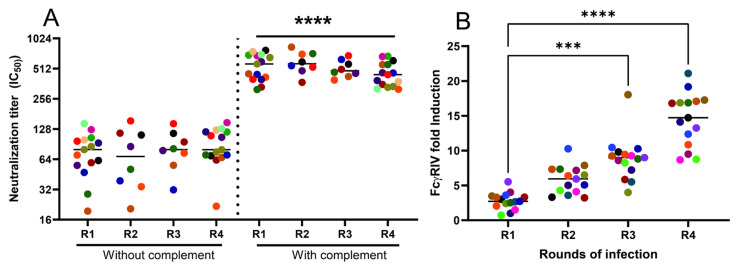
***Repeated exposures boost ADCC but not neutralizing antibody titers***. Neutralizing titer against HSV-1 in the absence or presence of 10% rabbit complement (**A**) and FcγRIV-activating ADCC-mediating antibodies using HSV-1 infected Vero cell lysates (**B**) were measured in serum obtained after each round of infection. Each individual mouse is color coded. The neutralization titer in the presence versus absence of complement was compared by paired *t*-test (*p* < 0.0001 after each round) and ADCC responses were compared relative to the response after primary infection (R1) by ANOVA with Friedman test for multiple comparisons (*** *p* < 0.001 and **** *p* < 0.0001).

The nAb titer was greater in the presence compared to in the absence of complement following each round of HSV-1 infection (Figure 4A). Repeated exposures, however, failed to boost either the complement-independent or complement-dependent neutralizing titers. In contrast, while primary infection elicited little or no detectable FcγRIV-activating antibodies, a biomarker of ADCC activity, there was a significant increase in response to subsequent viral exposures (Figure 4B).

**Figure 5:**
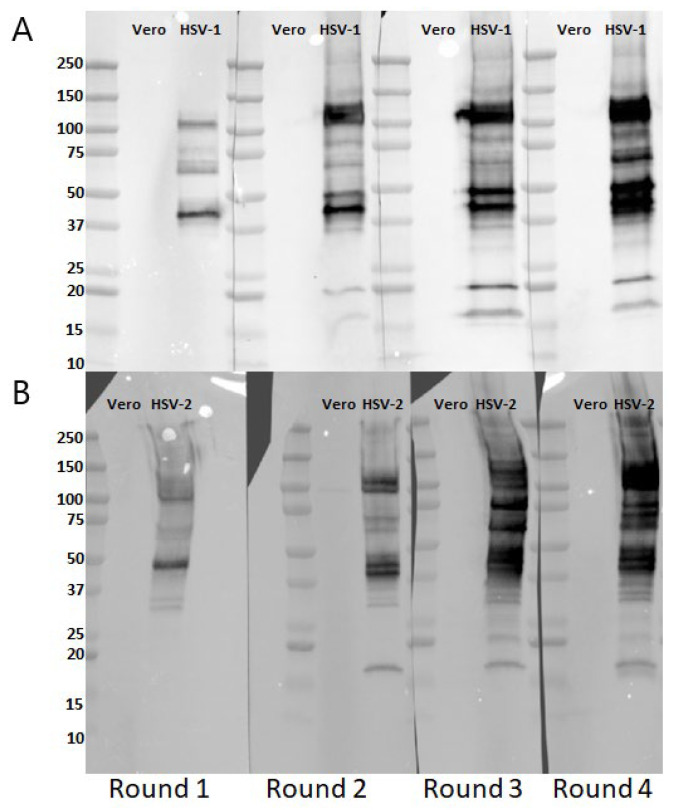
***Expansion in the breadth of viral antigenic targets recognized by immune serum following repeated viral exposures***. Western blots were performed with HSV-1-infected (**A**) or HSV-2 infected (**B**) and uninfected Vero cell lysates as the antigen. The blots were probed with immune serum from individual mice following each round of infection (R1–R4). Molecular weight markers are indicated on the left corresponding to the ladder (L). Shown are representative series of blots from one HSV-1 and one HSV-2 infected mouse at each time point.

Western blots with HSV infected compared to uninfected Vero cell lysates showed an increase in both the number and intensity of bands identified by immune serum with repeated viral exposures. The most predominant band identified in Immune serum from the initial round of infection primarily corresponded to gD (~MW 40 Kda) whereas bands corresponding in molecular weight to multiple other viral antigens were detected with immune serum harvested after subsequent viral exposures (Figure 5).

**Figure 6:**
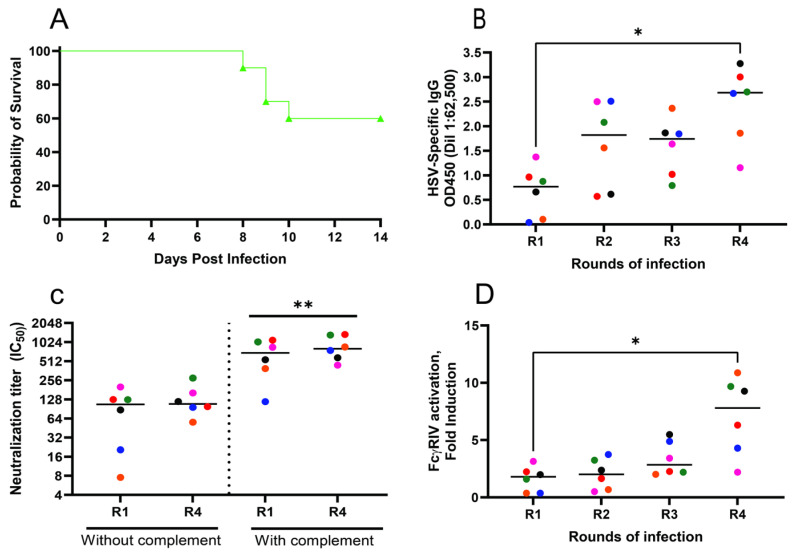
***Recurrent HSV-2 exposure boosts ADCC responses in mice.*** Mice (n = 10) were challenged monthly with 10^4^ pfu of HSV-2 (4674) and monitored for 14 days; survival after the first round is shown (**A**). Serum obtained 21 days after each round of infection was assayed for HSV-2 binding IgG by ELISA with infected cell lysates (**B**), neutralizing antibodies in the absence or presence of complement (**C**) or FcγRIV-activating ADCC-mediating antibodies (**D**). Results for each individual color-coded mouse are shown. The total HSV-2 binding IgG responses were compared at a serum dilution of 1:62,500 relative to primary response (R1) by ANOVA with correction for repeated measures (* *p* < 0.05). The differences in HSV-2 neutralizing titer in the presence or absence of complement for round 1 and round 4 were compared using paired *t*-tests (** *p* < 0.01) and the ADCC-mediating antibodies were compared relative to the response to primary (R1) infection by ANOVA with Freidman test for multiple comparisons (* *p* < 0.05).

***A similar evolution of antibody responses is observed in response to repeated HSV-2 exposures***. To determine if this recurrent sublethal intranasal infection model could also be used to assess changes in antibody responses to HSV-2, 10 mice (5 male and 5 female) were infected intranasally monthly with 10^4^ pfu of HSV-2 (4674). The majority survived the first round of infection (3/5 males and 3/5 females) and none succumbed to subsequent exposures (Figure 6A). The total HSV-2 binding IgG (measured by ELISA) was boosted in response to repeated exposures (*p* < 0.05 comparing R4 vs. R1, Figure 6B) but, as with HSV-1 (Figure 4A), there was no increase in the nAb titer, which again was augmented by the addition of complement (Figure 6C). However, there was a significant increase in ADCC-mediating antibodies (*p* < 0.05 comparing R4 vs. R1, Figure 6D) and western blots with HSV-2 infected cells as the target showed an increase in the intensity and breadth of antigenic targets (Figure 5B).

**Figure 7:**
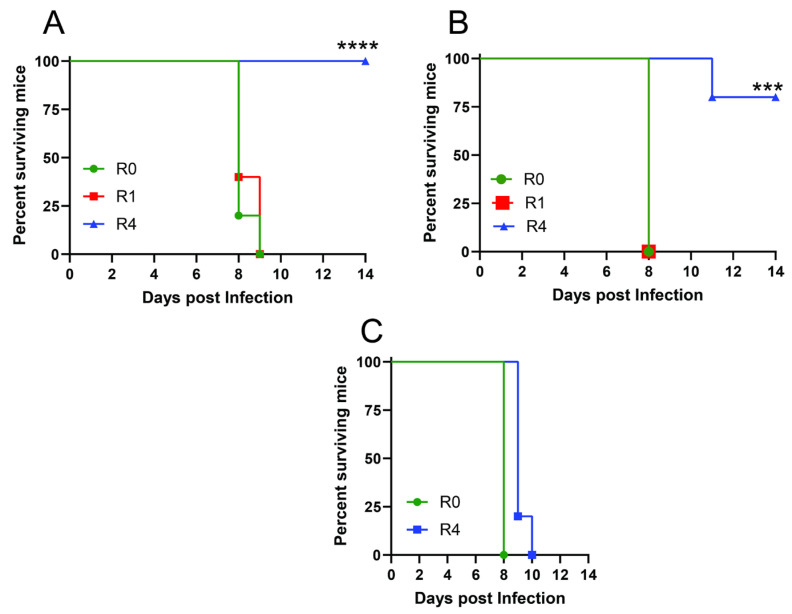
Immune serum obtained after four rounds of sublethal infection provides passive protection when transferred into naïve wild-type but not *Hvem* knockout mice. C57BL/6 mice received immune serum pooled from R0, R1 or R4 HSV-1 ((**A**), n = 10 mice per group in two independent experiments) or HSV-2 ((**B**), n = 5 mice per group) containing 750 μg of total IgG one day prior to skin challenge with an LD90 of the respective viruses. Survival was compared to mice that received R0 immune serum (**** *p* < 0.0001 for HSV-1 and *** *p* < 0.001 for HSV-2, Gehan-Breslow-Wilcoxon test). (**C**) Alternatively, the pooled immune serum (750 μg of total IgG) from R4 or R0 HSV-1 infected mice was passively transferred into *Hvem* knockout mice one day prior to skin challenge with an LD90 of HSV-1 (n = 5 mice per group).

***Immune serum with ADCC-mediating but not nAbs provides passive protection*.** Transfer of pooled immune serum collected after the fourth round of HSV-1 infection protected 10/10 mice from subsequent skin challenge with a lethal dose of HSV-1. Similarly. 4th round immune serum from HSV-2 sublethally-infected mice protected 4/5 mice from HSV-2 lethal skin challenge. In contrast, pooled immune serum collected after the first round of infection (containing neutralizing but not ADCC-mediating Abs) and serum obtained prior to infection (R0) failed to protect. (Figure 7A,B). However, protection was lost when the R4 HSV-1 pooled immune serum was transferred into *Hvem* knockout mice, which are impaired in mediating ADCC effector cell killing (Figure 7C) [12].

## 4. Discussion

Animal models that recapitulate clinical immune responses are critical tools for the development of vaccines, monoclonal antibodies and antivirals for the prevention or treatment of disease. Mice and guinea pigs are the more commonly used models as HSV replicates poorly in nonhuman primates. Mice are highly susceptible to both HSV-1 and HSV-2, but latency and spontaneous reactivations are rare, and efforts to induce reactivation are inconsistent. The guinea pig vaginal model offers several advantages as infection does not require pretreatment with medroxyprogesterone and spontaneous reactivations are observed more often. However, it is difficult to detect virus within reactivating lesions, knockout strains and reagents to study immune response are more limited, and the model has not proven predictive of vaccine trial outcomes [1]. For example, gD2/AS04, which failed to prevent disease in clinical trials [3], provided almost complete protection against primary HSV-1 or HSV-2 disease in the guinea pig model following vaginal challenge with the laboratory adapted strains HSV-1 (17 syn+) or HSV-2 (MS) and also protected against recurrences [17]. Similarly, the adjuvanted subunit vaccine comprised of gB and gD also provided near complete protection against intravaginal challenge with HSV-2 and reduced the frequency and severity of recurrences when previously infected guinea pigs were immunized but also failed in the clinical trials [2,18]. These vaccines were both designed to elicit neutralizing antibodies as the presumptive correlate of immune protection.

In prior studies, we modified the acute mouse model by challenging female and male mice on the skin with lethal doses of clinical isolates of HSV-1 or HSV-2 and found that this more stringent model recapitulated clinical vaccine trial results [11,12]. Building on that experience and to overcome the absence of a reproducible and predictive model of reactivation, we established a new model of recurrent sublethal HSV infections that recapitulates the antibody responses observed in patients with primary or longstanding HSV infection. We found that intranasal infection of female or male mice with 10^4^ pfu of clinical isolates of either HSV-1 or HSV-2 reproducibly resulted in seroconversion with 60–80% survival following the first round of infection and 100% survival following subsequent exposures. More importantly, the antibody responses mirrored what has been observed clinically. Using the same ELISA assay, we found that a single sublethal infection in mice elicited a neutralizing response with a titer of ~1:128, which is only one dilution different from the neutralization titer of 1:256 that we detected in women with primary HSV infection using samples available from placebo participants in the Herpevac gD/AS04 trial [5]. The addition of complement to the assay increased the neutralization activity of both human and mouse immune serum. Moreover, little or no ADCC-mediating antibodies were detected in response to acute infection in humans or mice. However, repeated sublethal exposures in mice resulted in the emergence of ADCC-mediating antibodies and an expansion in the number of viral proteins recognized on western blots, findings that paralleled what we observed in serum from an unrelated cohort of patients who had been HSV seropositive for at least five years (longstanding) [5]. The neutralizing antibodies persisted but were not boosted following repeated exposures in the mice again recapitulating what was observed comparing the neutralization titer in clinical samples from patients with acute versus longstanding HSV infection [5].

It is not known whether the emergence of ADCC-mediating antibodies in patients contributes to protection from clinical recurrences although clinical recurrences decline in frequency and severity in most patients over time. However, passive transfer studies using this new mouse model support this notion. The immune serum pooled from mice that had been repeatedly exposed to virus, which was replete with ADCC-mediating FcγRIV activating antibodies, passively protected naïve wild-type mice whereas serum pooled from mice that had only been infected once (acute), which had comparable neutralizing activity but no ADCC, failed to protect. The requirement for ADCC in mediating this protection was confirmed by its loss when the serum was transferred into *Hvem* knockout mice, which are impaired in ADCC effector cell killing [12]. The assertion that ADCC is important for protection against HSV disease is strengthened by a study of neonatal herpes which found that, after controlling for neutralizing titers, higher ADCC-mediating antibody levels were detected in the serum of mothers and their infants who had disease limited to the skin compared to those who developed disseminated neonatal disease [19]. This assertion is further supported by the failure of vaccines that elicit neutralizing but no ADCC to protect in clinical trials [4,5].

The emergence of ADCC responses following repeated infections was associated with an increase in the breadth of antigenic targets, again mirroring what we observed in our prior clinical studies [5]. We speculate that the anti-gD neutralizing antibody response elicited by primary infection facilitates this evolution by overcoming an immune evasion strategy mediated by gD and the co-signaling molecular switch molecule, HVEM, which is expressed by most immune cells. When gD binds HVEM, it blocks interactions between HVEM and its natural ligands, which results in a reduction in the generation of ADCC-mediating antibodies by yet unknown mechanisms [12]. Additionally, HVEM engagement with its natural ligands provides a second signal for effector cell killing [12] as further evidenced here by loss of passive protection when the immune serum was transferred into *Hvem* knockout mice. We speculate that the anti-gD neutralizing Abs remove this constraint and allow HVEM signaling to proceed, thus facilitating the generation of ADCC-mediating antibodies recognizing a broader repertoire of viral proteins. The targets of the ADCC-mediating responses have not yet been fully identified but include gB and other viral proteins [5] Using patient serum enriched for anti-gD or anti-gB antibodies or depleted in anti-gD and gB antibodies by fractionation on glycoprotein lectin columns, we found that the ADCC activity mapped to the anti-gB and gD/gB-depleted fractions but not the anti-gD fraction [15]. Studies in mice vaccinated with the single-cycle candidate vaccine strain deleted in gD (ΔgD-2), which protects by eliciting high titer ADCC-mediating antibodies, also identified gB and other viral antigens (but not gD) as targets of the ADCC response [9]. In particular, a monoclonal antibody targeting an epitope on domain IV of gB provided complete protection when mice were challenged with clinical isolates [20]. This new repeated low dose infection model provides the opportunity to identify the targets of ADCC responses elicited by recurrent infection and determine whether they or the same or different from those generated in response to ΔgD-2 or other candidate vaccines.

There are several limitations to this model including the fact that recurrent infection is not the same as reactivation from latency and that the intranasal route differs from the more common skin or mucosal routes of clinical exposure. However, the model recapitulates the antibody response to acute and chronic/longstanding infection and, in addition to identifying targets of the ADCC response, provides an opportunity to explore questions pertinent to HSV prevention and treatment. For example, while we did not observe any differences in the immune response in female compared to male mice, the study was not powered to detect sex differences, but future studies could address this. In addition, we focused only on quantifying neutralizing and ADCC-mediating responses, but the model can be expanded to explore the evolution of cell-mediated and innate immune responses taking advantage of wildtype and knockout mouse strains. Importantly, the model can also be applied to test whether candidate vaccines boost and/or elicit new types of immune responses in chronically (repeatedly challenged) infected mice, and, by passively transferring the immune serum, the protective efficacy of the vaccine responses.

It was surprising that the HSV-1 binding antibody titers were only transiently boosted after the second round of infection. Possibly, there is antibody affinity maturation with successive rounds of exposure as reflected by the increase in ADCC-mediating antibodies and expansion in antigenic repertoire but a concomitant loss in lower affinity binding antibodies. The response to HSV-2 differed as both the total HSV-binding and ADCC-mediating response continued to increase with successive exposures reaching significance only after the fourth round. Further studies are needed to test this notion that the discordance between the total HSV-1 binding antibody titer and increase in ADCC reflects affinity maturation and why the response to HSV-1 and HSV-2 differs in this model.

In summary, results obtained from this new model highlight the importance of ADCC as a correlate of protection as only the immune serum with ADCC activity, but not immune serum with equivalent neutralizing but no ADCC activity, provided passive protection. The findings do not preclude the possibility that higher titer neutralizing responses and/or those targeting additional antigens may also prove protective. The model provides the opportunity to test this question and to identify thresholds and targets of both neutralizing and ADCC antibodies needed for protection.

## Data Availability

All data supporting the findings of this study are available on request.

## References

[1] Aschner C.B., Herold B.C. (2021). Alphaherpesvirus Vaccines. Curr. Issues Mol. Biol..

[2] Corey L., Langenberg A.G., Ashley R., Sekulovich R.E., Izu A.E., Douglas J.M., Handsfield H.H., Warren T., Marr L., Tyring S. (1999). Recombinant glycoprotein vaccine for the prevention of genital HSV-2 infection: Two randomized controlled trials. Chiron HSV Vaccine Study Group. JAMA.

[3] Belshe R.B., Leone P.A., Bernstein D.I., Wald A., Levin M.J., Stapleton J.T., Gorfinkel I., Morrow R.L., Ewell M.G., Stokes-Riner A. (2012). Efficacy results of a trial of a herpes simplex vaccine. N. Engl. J. Med..

[4] Kohl S., Charlebois E.D., Sigouroudinia M., Goldbeck C., Hartog K., Sekulovich R.E., Langenberg A.G., Burke R.L. (2000). Limited antibody-dependent cellular cytotoxicity antibody response induced by a herpes simplex virus type 2 subunit vaccine. J. Infect. Dis..

[5] Mahant A.M., Guerguis S., Blevins T.P., Cheshenko N., Gao W., Anastos K., Belshe R.B., Herold B.C. (2022). Failure of Herpes Simplex Virus Glycoprotein D Antibodies to Elicit Antibody-Dependent Cell-Mediated Cytotoxicity: Implications for Future Vaccines. J. Infect. Dis..

[6] Hoshino Y., Pesnicak L., Dowdell K.C., Lacayo J., Dudek T., Knipe D.M., Straus S.E., Cohen J.I. (2008). Comparison of immunogenicity and protective efficacy of genital herpes vaccine candidates herpes simplex virus 2 dl5-29 and dl5-29-41L in mice and guinea pigs. Vaccine.

[7] Dropulic L.K., Oestreich M.C., Pietz H.L., Laing K.J., Hunsberger S., Lumbard K., Garabedian D., Turk S.P., Chen A., Hornung R.L. (2019). A Randomized, Double-Blinded, Placebo-Controlled, Phase 1 Study of a Replication-Defective Herpes Simplex Virus (HSV) Type 2 Vaccine, HSV529, in Adults With or Without HSV Infection. J. Infect. Dis..

[8] Wang K., Dropulic L., Bozekowski J., Pietz H.L., Jegaskanda S., Dowdell K., Vogel J.S., Garabedian D., Oestreich M., Nguyen H. (2021). Serum and Cervicovaginal Fluid Antibody Profiling in Herpes Simplex Virus-Seronegative Recipients of the HSV529 Vaccine. J. Infect. Dis..

[9] Petro C.D., Weinrick B., Khajoueinejad N., Burn C., Sellers R., Jacobs W.R., Herold B.C. (2016). HSV-2 DeltagD elicits FcgammaR-effector antibodies that protect against clinical isolates. JCI Insight.

[10] Burn Aschner C., Knipe D.M., Herold B.C. (2020). Model of vaccine efficacy against HSV-2 superinfection of HSV-1 seropositive mice demonstrates protection by antibodies mediating cellular cytotoxicity. npj Vaccines.

[11] Burn C., Ramsey N., Garforth S.J., Almo S., Jacobs W.R., Herold B.C. (2018). A Herpes Simplex Virus (HSV)-2 Single-Cycle Candidate Vaccine Deleted in Glycoprotein D Protects Male Mice From Lethal Skin Challenge With Clinical Isolates of HSV-1 and HSV-2. J. Infect. Dis..

[12] Burn Aschner C., Loh L.N., Galen B., Delwel I., Jangra R.K., Garforth S.J., Chandran K., Almo S., Jacobs W.R., Ware C.F. (2020). HVEM signaling promotes protective antibody-dependent cellular cytotoxicity (ADCC) vaccine responses to herpes simplex viruses. Sci. Immunol..

[13] Kao C.M., Goymer J., Loh L.N., Mahant A., Burn Aschner C., Herold B.C. (2020). Murine Model of Maternal Immunization Demonstrates Protective Role for Antibodies That Mediate Antibody-Dependent Cellular Cytotoxicity in Protecting Neonates From Herpes Simplex Virus Type 1 and Type 2. J. Infect. Dis..

[14] Wang Y., Subudhi S.K., Anders R.A., Lo J., Sun Y., Blink S., Wang Y., Wang J., Liu X., Mink K. (2005). The role of herpesvirus entry mediator as a negative regulator of T cell-mediated responses. J. Clin. Investig..

[15] Mahant A.M., Trejo F.E., Aguilan J.T., Sidoli S., Permar S.R., Herold B.C. (2023). Antibody attributes, Fc receptor expression, gestation and maternal SARS-CoV-2 infection modulate HSV IgG placental transfer. iScience.

[16] Visciano M.L., Mahant A.M., Pierce C., Hunte R., Herold B.C. (2021). Antibodies Elicited in Response to a Single Cycle Glycoprotein D Deletion Viral Vaccine Candidate Bind C1q and Activate Complement Mediated Neutralization and Cytolysis. Viruses.

[17] Bourne N., Bravo F.J., Francotte M., Bernstein D.I., Myers M.G., Slaoui M., Stanberry L.R. (2003). Herpes simplex virus (HSV) type 2 glycoprotein D subunit vaccines and protection against genital HSV-1 or HSV-2 disease in guinea pigs. J. Infect. Dis..

[18] Burke R.L. (1991). Development of a herpes simplex virus subunit glycoprotein vaccine for prophylactic and therapeutic use. Rev. Infect. Dis..

[19] Kohl S., West M.S., Prober C.G., Sullender W.M., Loo L.S., Arvin A.M. (1989). Neonatal antibody-dependent cellular cytotoxic antibody levels are associated with the clinical presentation of neonatal herpes simplex virus infection. J. Infect. Dis..

[20] Kuraoka M., Aschner C.B., Windsor I.W., Mahant A.M., Garforth S.J., Kong S.L., Achkar J.M., Almo S.C., Kelsoe G., Herold B.C. (2023). A non-neutralizing glycoprotein B monoclonal antibody protects against herpes simplex virus disease in mice. J. Clin. Investig..

